# Phylogeographic Reconstruction to Trace the Source Population of Asian Giant Hornet Caught in Nanaimo in Canada and Blaine in the USA

**DOI:** 10.3390/life14030283

**Published:** 2024-02-20

**Authors:** Alexa Freeman, Xuhua Xia

**Affiliations:** 1 Department of Biology, University of Ottawa, Ottawa, ON K1N 9A7, Canada; 2 Ottawa Institute of Systems Biology, University of Ottawa, Ottawa, ON K1H 8M5, Canada

**Keywords:** *Vespa mandarinia*, invasive species, conservation biology, DNA barcoding, phylogeography, geophylogeny

## Abstract

The Asian giant hornet, *Vespa mandarinia*, is an invasive species that could potentially destroy the local honeybee industry in North America. It has been observed to nest in the coastal regions of British Columbia in Canada and Washington State in the USA. What is the source population of the immigrant hornets? The identification of the source population can shed light not only on the route of immigration but also on the similarity between the native habitat and the potential new habitat in the Pacific Northwest. We analyzed mitochondrial COX1 sequences of specimens sampled from multiple populations in China, the Republic of Korea, Japan, and the Russian Far East. *V. mandarinia* exhibits phylogeographic patterns, forming monophyletic clades for 16 specimens from China, six specimens from the Republic of Korea, and two specimens from Japan. The two mitochondrial COX1 sequences from Nanaimo, British Columbia, are identical to the two sequences from Japan. The COX1 sequence from Blaine, Washington State, clustered with those from the Republic of Korea and is identical to one sequence from the Republic of Korea. Our geophylogeny, which allows visualization of genetic variation over time and space, provides evolutionary insights on the evolution and speciation of three closely related vespine species (*V. tropica*, *V. soror,* and *V. mandarinia*), with the speciation events associated with the expansion of the distribution to the north.

## 1. Introduction

The genus *Vespa*, natively distributed in tropical, subtropical, and temperate Asia, features several invasive hornet species [1,2,3]. The accidental introduction of *V. velutina* to Europe [4] severely affected European apiculture, leading to tens of millions of dollars in management costs [5]. *V. mandarinia* is the largest species within the genus known to cause severe damage to apiculture [6,7] and human deaths [7,8]. While the Asian honey bee (*Apis cerana*) can sense and respond defensively against *V. mandarinia*, the European honey bee (*Apis mellifera*), which has come into the range of *V. mandarinia* only recently, cannot [7,9,10]. Multiple stings by *V. mandarinia* can be lethal to humans, with a mean number of only 59 stings causing death [8]. Note that vespine hornets have smooth singers and can sting the victim repeatedly in quick succession, in contrast to honeybees that have barbed stingers and can sting only once before they die.

Although the native distribution of *V. mandarinia* is in Asian countries and the Russian Far East [11,12,13], several individuals of the species have been observed in coastal regions of Washington State [10,14] and British Columbia since 2019 [15,16,17]. The USA and Canadian sites where the Asian giant hornets were found have a distance of about 95 km [10] and might have been brought to these sites by the same shipping route. However, they have distinct mitochondrial genomes and might come from different source populations in Asia [10].

These potentially harmful immigrants raised several questions. First, where is their source population in Asia? Second, could the few founders survive, reproduce, and establish a viable population that propagates itself in North America? The second question is often addressed with habitat niche modeling [14,15,18]. However, addressing the first question would shed light on the second. If the invading Asian giant hornets were from a population in a habitat similar to that in North America, then the invaders likely would survive and reproduce. Observations in British Columbia suggested strongly that *V. mandarinia* could survive and reproduce in North America. An underground nest of Asian giant hornets was found around Robin’s Park on 18 September 2019 [15,16,17]. All of the hornets from the nest, including the queen, were transferred out of the nest, and the nest was destroyed by the utilization of carbon dioxide [15,16,17]. These observations are consistent with the observation of *V. mandarinia* in Blaine, WA [10], reinforcing the inference that the pest has the potential to survive and reproduce in the coastal regions of British Columbia and Washington State [14].

In this report, we aim to address the first question, i.e., the identification of the source population of those *V. mandarinia* individuals invading Canada and the USA. This can be achieved by using DNA barcoding data that includes not only DNA barcodes but also geographic coordinates or sampling locations from which geographic coordinates can be approximately derived. Such DNA barcoding data for *V. mandarinia* are available for multiple populations in China, the Republic of Korea, Japan, and the Russian Far East. The rationale and the operational protocol are simple. A source population needs to fulfill two criteria. First, the mitochondrial COX1 sequences from the source population should be most closely related (ideally identical) to those found in Canada and the USA. Second, the COX1 sequences from other *V. mandarinia* populations are more different than the source population from those found in Canada and the USA. This approach is similar to identifying illegally hunted whale species in the whale meat market [19]. If the DNA in a piece of whale meat is identical to that of the protected humpback whale but different from all other known whale species, then the whale meat is from an illegally hunted humpback whale. However, identifying a source population is more difficult than species identification.

*V. mandarinia* is a widely distributed temperate species [6,7,13,20]. If the species is genetically homogeneous so that populations in different regions all share the same genotype or the same mixture of genotypes, then the above approach for identifying the source population of the *V. mandarinia* individuals invading Canada and the USA would not work. However, there are indications of genetic differentiation among *V. mandarinia* populations revealed in previous studies [10,21]. Some of the *V. mandarinia* lineages were even given subspecies status [6]. This suggests the possibility of identifying the source population by using genetic markers such as mitochondrial COX1 sequences, as had previously been conducted with *V. velutina* in Europe [22]. The approach has been taken to identify the source population for an invading individual in *V. crabro* [1]. A preliminary identification has already been made [10] to identify the source of the *V. mandarinia* individuals caught in Canada and the USA, with a phylogenetic tree including five *V. mandarinia* specimens (the Canada and USA specimens plus one specimen each from China, Japan, and the Republic of Korea). Based on the comparison of mitochondrial genes, the Canadian specimen was found to be most similar to the Japanese specimen with an evolutionary distance of 0.0012, and the USA specimen most similar to the Republic of Korea specimen with a distance of 0.0004 [10]. However, it is possible that *V. mandarinia* populations in other Chinese populations may have a distance even smaller than those reported between the Canadian and the Japanese specimens or between the USA and the South Korean specimens.

We compiled and analyzed mitochondrial COX1 sequences of specimens sampled from multiple populations in China, the Republic of Korea, Japan, and the Russian Far East. *V. mandarinia* populations exhibit genetic differentiation among different geographic regions, with specimens from China, the Republic of Korea, and Japan forming distinct clades. The two mitochondrial COX1 sequences from Nanaimo, British Columbia, are identical to the two sequences from Japan (from Fukuoka and Yamaguchi, respectively). The COX1 sequence from Blaine, Washington State, is identical to one of six sequences from the Republic of Korea.

## 2. Material and Methods

All sequences were downloaded from GenBank except for four sequences downloaded from the DNA barcoding Bold System [23] with sample IDs BIOUG26171-B05, BIOUG24885-F10, NIBGE HYM-01572, and NIBGE HYM-01001). The criteria for inclusion are (1) that the original publication contains a description of sampling locations from which approximate latitude and longitude values can be derived and (2) that the sequence is not a fragment of a longer sequence sampled from the same location. For example, the sequence with GenBank accession MZ165595 was included, but MZ165596 was not because the latter is identical to a segment within the former, and the two were sampled at the same location, i.e., Ruili, China, near the border between China and Myanmar.

For specimens with complete mitochondrial genomes, the COX1 sequences were extracted from the GenBank file with DAMBE [24]. Four COX1 sequences from *V. tropica* and three COX1 sequences from *V. soror* were used as outgroups. Previous studies [20,21,25,26] have shown *V. soror* to be the closest relative of *V. mandarinia* within the genus. A total of 36 COX1 sequences were included.

One included sequence (Accession KR059904) requires some clarification. It is the first *V. mandarinia* mitochondrial genome sequenced in China [27] and has often been used as a reference genome to assemble mitochondrial genomes [10]. However, its sampling location has not been recorded in publications. We emailed Dr. Shu-Jun Wei and learned that the specimen was collected in Chengde City in Hebei Province in Northern China, which yields approximate latitude and longitude values of 40.94 and 117.99, respectively. The GenBank accession number, species name, geographic locations in latitude and longitude, COX1 length, and GC% are listed in Table 1.

The resulting *COX1* sequences were aligned using MAFFT [28] with the most accurate LINSI option (‘–localpair’ and ‘–maxiterate = 1000’). For phylogenetic reconstruction, the GTR + Γ model was used with four discrete rate categories for approximating a continuous gamma distribution [29]. This model was chosen based on the information-theoretic index AIC (Table 2) and the likelihood ratio tests (Table 3) [30,31] among the nested HKY [32], TN93 [33], and GTR [34,35] models with or without the discrete gamma distribution to accommodate rate heterogeneity in substitution rate among sites.

The tree log-likelihood (lnL) was obtained with PhyML v3.3 [36]. The tree improvement option (‘-s’) was set to ‘BEST’ (best of NNI and SPR search). The ‘-o’ option was set to ‘tlr’, which optimizes the topology, the branch lengths, and rate parameters. The phylogenetic trees reported in the Results section were from PhyML reconstruction with GTR + Γ, which has the smallest AIC (Table 2) and fits the data significantly better than the alternative models (Table 3). MAFFT and PhyML are included in DAMBE and called to analyze sequences with a consistent user interface. 

The phylogeographic analysis aims to visualize genetic variation over time and space. A geophylogeny combines a phylogenetic tree together with the geographic coordinates of sampling locations so that one can visualize phylogenetic relationships and biogeographic distribution of evolutionary lineage. We used PGT software version 1.0.0 [37] to generate geophylogenies for visualization. PGT makes use of both Google Maps and Microsoft Bing Maps with regular map view and satellite terrain view.

Because the limited number of sequenced specimens does not truly reflect the global distribution of *V. mandarinia*, we have also downloaded the geographic distribution of 3165 recorded presences of Asian giant hornets from GBIF [38]. We excluded two dubious records with geographic coordinates that happen to be the geographic centroid of China and India, respectively. There has never been any *V. mandarinia* recorded in such two locations. It is likely that they do not have actual geographic coordinates but were given the centroid of the country as placeholders of geographic coordinates.

## 3. Results

### 3.1. Identification of the Source Population for the Specimens from Canada and the USA 

**Figure 1:**
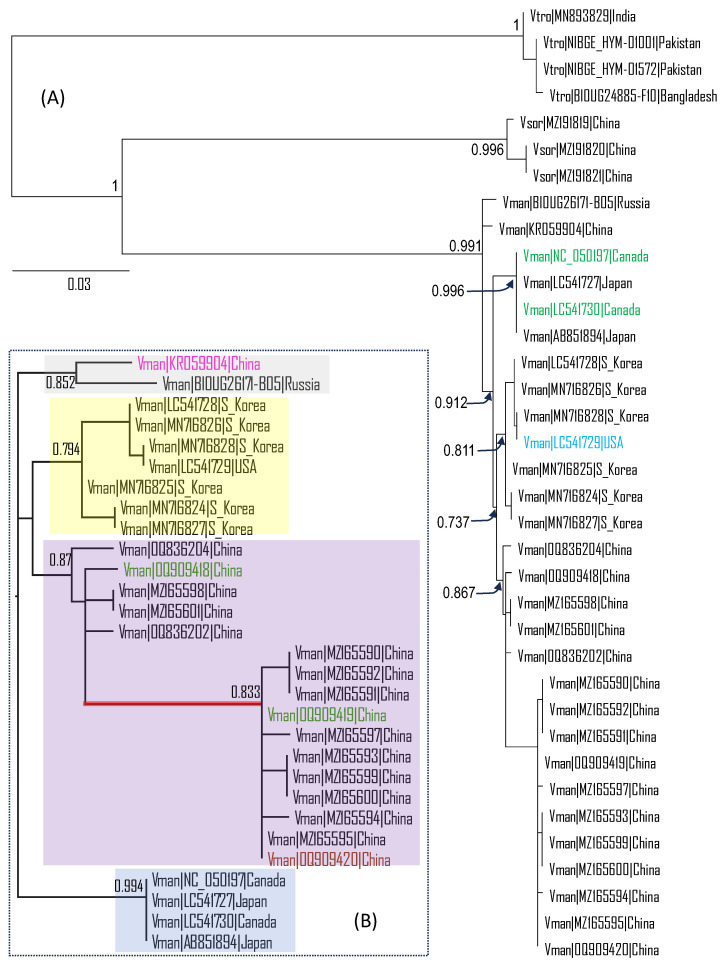
The PhyML tree with support values at key internal nodes. The taxon name is in the form of “Species_name|GenBank accession|Country”. Vman—*Vespa mandarinia*; Vsor—*V. soror*; Vtro—*V. tropica*. (**A**) The unrooted phylogeny is displayed by midpoint rooting using the FigTree [39]. The COX1 sequence from the USA specimen (colored blue) is identical to the one from the Republic of Korea (S_Korea, accession MN716828). The two COX1 sequences from Canada (colored green) are identical to the two COX1 sequences of specimens sampled from Japan. (**B**) An unrooted phylogeny of *V. mandarinia* was reconstructed independently without the two outgroup species. Only four specimens from China are not from Yunnan Province: the pink-colored from Northeastern China, the two green-colored from Guangdong Province (Southeastern China), and the purple-colored from Sichuan Province (Western China). We designate the four lineages in (**B**) as the Japan lineage (shaded blue), the China lineage (shaded purple), the Korean lineage (shaded yellow), and the Northeastern Asia lineage (shaded grey).

The three *Vespa* species, *V. tropica*, *V. soror*, and *V. mandarinia*, are well differentiated genetically from each other (Figure 1A). There is also clear but less pronounced geographic differentiation within *V. mandarinia*, forming monophyletic clades for 16 specimens from China (Yunnan, Guangdong, and Sichuan provinces), six specimens from the Republic of Korea, and two specimens from Japan (Figure 1A). This genetic differentiation over space facilitates the identification of the source populations to which the invading individuals found in Canada and the USA belong. If all *V. mandarinia* populations are genetically homogeneous, then there would be no chance of identifying a specific source population for any invading individual.

The two mitochondrial COX1 sequences of the *V. mandarinia* specimens from Nanaimo, British Columbia, are identical to each other and to the two sequences from Japan (which are also identical to each other). The collection sites of the two in Japan have a straight-line distance of 120 km between Fukuoka and Yamaguchi. The lack of genetic variation between the two *V. mandarinia* specimens suggests that they were recent descendants of a common ancestor. In contrast, the South Korean specimens exhibited substantial genetic variation, as exemplified by the branch lengths in Figure 1. The COX1 sequence from Blaine, Washington State, clustered with those from the Republic of Korea and is identical to one sequence from the Republic of Korea (GenBank accession MN716828, Figure 1A). Thus, one may tentatively infer that the source populations for the Canada and the USA specimens of *V. mandarinia* were most likely in Japan and the Republic of Korea, respectively. However, this inference is not strong because there is no specimen sampled along the coastal regions of East China opposite Japan and the Republic of Korea. It is possible that specimens from such coastal regions may also have COX1 sequences identical to the Canada and the USA specimens, respectively. Thus, identifying a specimen to its source population is much more difficult than identifying a piece of whale meat to whale species.

The unrooted phylogeny (Figure 1B) shows the genetic variation within *V. mandarinia.* It is reconstructed with PhyML without sequences from the two outgroup species, but the tree is nearly identical to the subtree of *V. mandarinia* sequences in Figure 1A, indicating the robustness of the phylogenetic relationship. Four geographic lineages within *V. mandarinia* are clearly distinguishable in Figure 1B: the Japan lineage (two specimens from Japan plus two invasive individuals caught in Canada, shaded blue), the China lineage (16 specimens, shaded purple), the Korean lineage (six specimens in the Republic of Korea plus one invasive individual caught in the USA, shaded yellow) and the Northeastern Asia lineage (two specimens, one from the Russian Far East and the other from Northern China, shaded grey) The mainland China lineage is separated into two subgroups by the long red branch (Figure 1B). What is remarkable is that these two genetically different subgroups are not geographically separated. Each subgroup has colonized Yunan Province and Guangdong Province. Thus, distinct lineages of *V. mandarinia* appeared to have evolved sympatrically. 

Confusion remains as to subspecies designation within *V. mandarinia*. At least five subspecies were previously listed [7] but subsequently reduced to three [40] or not recognized at all [12]. The Global Biodiversity Information Facility [38] lists *V. m. japonica*, *V. m. magnifica*, and *V. m. nobilis*, with distribution information, *V. m. japonica*, and *V. m. magnifica* correspond to the blue and purple lineages in Figure 1B. No sequence data is available for *V. m. nobilis* in Taiwan. The grey and yellow lineages in Figure 1B may also be considered for subspecies status, given their phylogenetic relationships.

### 3.2. Phylogeographic Patterns of V. mandarinia and the Two Outgroup Species

**Figure 2:**
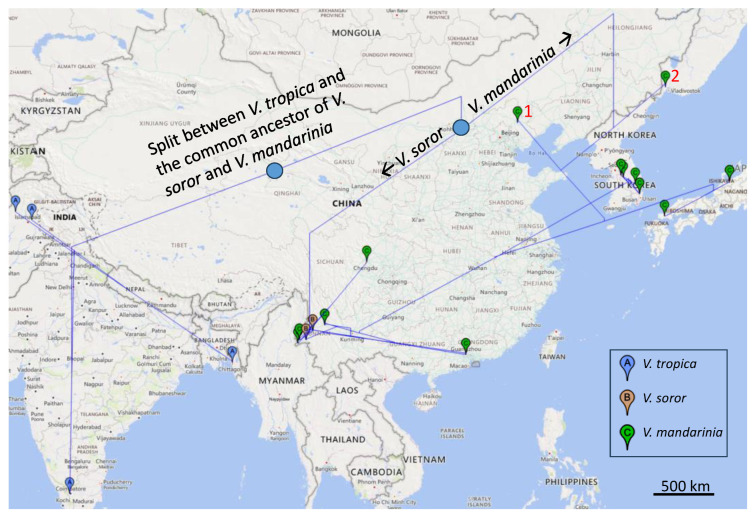
Geophylogeny of the specimens from the three *Vespa* species generated from the PGT program [37]. The species legend is in the inset. The vertical lines are branch lengths of the tree. The two specimens indicated by red-colored 1 and 2 are closely related and may represent a unique Northeastern Asia lineage. The geophylogeny in Figure 2 highlights a strong insufficiency in sample collection in China. There is no specimen sequenced in the coastal regions of East China facing Japan and the Republic of Korea across the East China Sea and Yellow Sea, respectively. However, according to the Global Biodiversity Information Facility [38], our 16 specimens in China should belong to the subspecies *V. mandarinia magnifica*, whose distribution includes the eastern coast of China. Therefore, there should be little doubt that the Canadian specimen was from Japan and the USA specimen was from the Republic of Korea.

The distribution of genetic variation over both time and space is better visualized through geophylogeny (Figure 2), which superimposes a phylogenetic tree over a geographic region to summarize the genetic variation of evolutionary lineages over time and space [37]. Of the two outgroup species, *V. tropica* is widely distributed in tropical Asia [7,13,41]. Although *V. tropica* may be found occasionally in temperate regions, its nest size is much smaller as a consequence of reduced availability of food (i.e., eggs and larvae of polistine wasps) [7]. *V. soror*, the southern giant hornet, inhabits the tropical and subtropical regions of Southeastern Asia, including China [6,7,13,20]. The speciation initiated with the tropical *V. tropica* splitting from the common ancestor of *V. soror* and *V. mandarinia* (Figure 2). This common ancestor likely moved from the tropical to subtropical habitats, with *V. soror* remaining in the subtropical region but *V. mandarinia* invading the temperate region (Figure 2). If we may interpret the geophylogeny (Figure 2) liberally, then the ancestral lineages of *V. mandarinia* first inhabited the northern temperate region, represented by the early lineages marked with red-colored 1 in Northern China) and 2 in the Russian Far East (Figure 2). From these northern lineages, the Japanese lineage of *V. mandarinia* (Figure 1 and Figure 2). The South Korean clade and the Southern China clade form a monophyletic clade (Figure 1 and Figure 2). Thus, the geographic expansion and the association appear to be from the tropical *V. tropica* to the subtropical *V. soror* and to the temperate *V. mandarinia.* However, the differentiation within *V. mandarinia* may require confirmation of the rooting position with more data. Only two vespine wasps (*V. mandarinia* and *V. crabro*) have expanded as far north as Hokkaido in Japan, while all other vespine wasps, such as *V. tropica*, are restricted to southern Japan [6,7]. The adaptation of *V. mandarinia* to a temperate climate would increase its chance for survival and reproduction in the coastal regions of British Columbia and Washington State.

**Figure 3:**
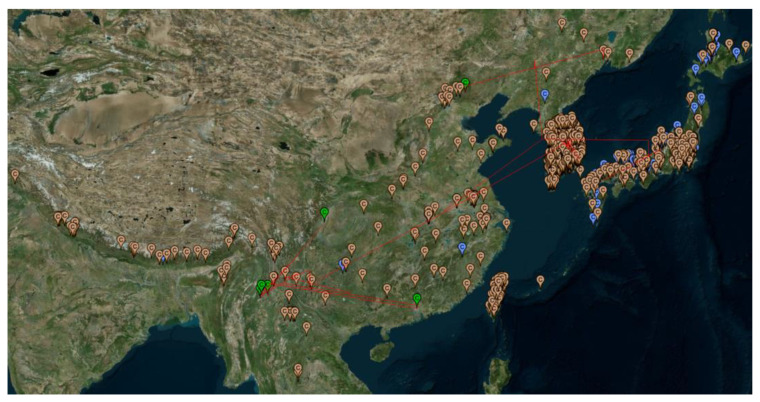
Geophylogeny of *V. mandarinia* specimens showing four phylogenetic clades (Northern Asia, Japanese, the Republic of Korea, and Chinese clades) in different geographic regions, together with unsequenced specimens from GBIF [38]. Green pins: sequenced specimens in the geophylogeny. Blue pins: the recorded presence of *V. mandarinia* before the year 2000. Orange pins: recorded presence from the year 2000. Taiwan recorded multiple presences of *V. mandarinia* both before and after 2000, but those blue pins before 2000 were obscured by the orange pins. Generated from PGT [37]. The letter C in the pins has no meaning.

To better visualize the natural habitats of *V. mandarinia*, we have generated a geophylogeny for *V. mandarinia* specimens alone (Figure 3) and supplemented the geophylogeny with the recorded presence of *V. mandarinia* compiled in the GBIF database [38]. It is clear that *V. mandarinia* occupies a wide temperate zone, but its natural habitats tend to be humid and forested, which resembles the environment in the coastal regions of British Columbia in Canada and Washington States in the USA. If all the recorded sites of *V. mandarinia* were represented by sequenced specimens, which is likely achievable in the next 10 years, then one would be able to identify highly accurately the source population of any invasive individuals of *V. mandarinia*.

The relatively few sites recorded in mainland China and India, especially before 2000 (Figure 3), could have two explanations. First, there was little research effort in sampling and documenting the distribution of *V. mandarinia.* Second, the species is indeed much less abundant on the mainland than on the islands. The dramatically increased number of recorded presences after the year 2000 relative to those before the year 2000 (Figure 3) suggests that the first hypothesis is more likely than the second. 

We should emphasize here that more data are needed to establish the rooting position for *V. mandarinia* (Figure 1). With the general trend of the expansion of the *Vespa* species from tropical to temperate regions (Figure 2), it seems odd that the two northern specimens of *V. mandarinia* should be the closest to the root (Figure 1 and Figure 2).

## 4. Discussion

The tentative identification of *V. mandarinia* populations in Japan and the Republic of Korea as the source populations of the Canadian and USA specimens, respectively (Figure 1), facilitates downstream research and monitoring. First, Canadian and the USA border services on invasive insect species should focus more on incoming cargo from Japan and the Republic of Korea than from elsewhere. Second, in terms of habitat modeling of *V. mandarinia*, the input habitat data from Japan and the Republic of Korea should carry more weight than those from elsewhere. Third, any invading individuals of *V. mandarinia* that are genetically distinct from the Japanese and South Korean populations of *V. mandarinia* would suggest a new source population. Tracing an invading individual to its source population is crucial in understanding its habitat requirement for survival and reproduction. Different *V. mandarinia* lineages could have different habitat requirements, so it could be misleading to model the habitat requirement of invading individuals from a Japanese *V. mandarinia* population by using the habitat requirement of certain Chinese *V. mandarinia* populations as input.

The biogeographic distribution of a species is determined mainly by three factors: (1) the dispersal ability of the species, (2) the ability to survive and reproduce in the new environment, and (3) the time needed for dispersal and adaptation to colonize a new habitat. Many failed experiments that introduced animals into seemingly suitable new habitats or reintroduced zoo-raised animals into the natural habitat of their ancestors highlight the importance of the ability to survive and reproduce in a new environment.

Vespine hornets appear to be highly capable of survival and reproduction in new environments and include multiple invasive species [2,3]. Although *V. velutina* is perhaps the most notorious of vespine wasps [4,5,21,26,42,43], *V. mandarinia* is also known for attacking honeybee hives en masse and incurring high economic losses [6,7]. It is also well documented that Asian giant hornets carry multiple viruses with yet unknown implications on environmental health [44]. Furthermore, it takes an average of only 59 stings by *V. mandarinia* to cause human death [8]. Thus, it is important to prevent *V. mandarinia* from spreading to North America. Accurate identification of the source population of invading individuals of *V. mandarinia* would provide key information in stopping the spread of the invasive species.

DNA barcoding has been used for the identification of invasive species, especially when samples represent eggs, immature larvae, or poorly preserved specimens [45,46]. The geographic information associated with DNA-barcoded specimens has contributed to the elucidation of colonization and speciation of Hawaiian wingless katydids [47]. However, to identify the source population of an invading individual, extensive sampling and sequencing information is needed. For example, the identification of the Canadian and USA specimens to their source populations would have been more accurate and confident if we had sequence information from specimens sampled along the coastal populations of *V. mandarinia* in East China. One needs to DNA-barcode not only species but also natural populations, ideally before human-mediated long-distance transportation obliterates the natural biogeographic patterns. Another problem with the current DNA barcoding data is that only a partial sequence of the mitochondrial COX1 gene is used as a DNA barcode. Such a short sequence increases the chance of the COX1 sequence from an invasive individual matching DNA barcodes from multiple populations, rendering it impossible to pinpoint which is the source population.

## 5. Conclusions

The *V. mandarinia* individuals invading British Columbia in Canada and Washington State in the USA have mitochondrial COX1 sequences identical to those of *V. mandarinia* specimens collected in Japan and the Republic of Korea, respectively. They are distinct from specimens sampled in various locations in mainland China. However, for DNA barcoding data to be practically useful for identifying invasive individuals to their source populations, longer DNA barcodes (e.g., the entire mitochondrial genome) and specimens from all representative populations should be included in relevant databases. Our phylogeographic results serve as a basis for inferring speciation and biological adaptation in vespine species.

## Figures and Tables

**Table 1:** GenBank accession (ACCN), species, latitude and longitude, sequence length of the mitochondrial COX1 gene (L_COX1_), and GC%. Four sequences without a GenBank accession were listed with the sample ID of the BOLD System [23].

ACCN/Sample ID	Species	Latitude	Longitude	L_COX1_	GC%
AB851894	*V. mandarinia*	36.77	137.47	655	30.84
BIOUG26171-B05	*V. mandarinia*	43.35	131.57	588	29.76
KR059904	*V. mandarinia*	40.94	117.99	1533	29.88
LC541727	*V. mandarinia*	34.18	131.47	1536	29.88
LC541728	*V. mandarinia*	37.06	127.70	1536	30.08
LC541729	*V. mandarinia*	48.99	−122.75	1536	30.01
LC541730	*V. mandarinia*	49.18	−123.94	1536	29.88
MN716824	*V. mandarinia*	36.60	128.78	658	31.16
MN716825	*V. mandarinia*	35.84	129.22	658	31.31
MN716826	*V. mandarinia*	35.84	129.22	658	31.16
MN716827	*V. mandarinia*	37.22	127.48	658	31.16
MN716828	*V. mandarinia*	37.22	127.48	658	31.00
MZ165590	*V. mandarinia*	25.11	99.17	766	31.59
MZ165591	*V. mandarinia*	25.11	99.17	777	31.40
MZ165592	*V. mandarinia*	25.11	99.17	778	31.36
MZ165593	*V. mandarinia*	24.37	97.96	818	30.81
MZ165594	*V. mandarinia*	24.37	97.96	791	31.23
MZ165595	*V. mandarinia*	24.08	97.82	854	31.26
MZ165597	*V. mandarinia*	25.11	99.17	762	31.36
MZ165598	*V. mandarinia*	24.36	98.57	766	31.59
MZ165599	*V. mandarinia*	24.37	97.96	790	31.27
MZ165600	*V. mandarinia*	25.11	99.17	793	31.40
MZ165601	*V. mandarinia*	24.36	98.57	772	31.48
OQ836202	*V. mandarinia*	25.61	100.27	1536	29.82
OQ836204	*V. mandarinia*	25.61	100.27	1536	29.95
OQ909418	*V. mandarinia*	23.17	113.27	666	31.23
OQ909419	*V. mandarinia*	23.17	113.27	658	31.16
OQ909420	*V. mandarinia*	30.67	104.14	657	31.35
NC_050197	*V. mandarinia*	49.18	−123.94	1536	29.88
MZ191819	*V. soror*	25.11	99.17	783	30.40
MZ191820	*V. soror*	24.36	98.57	834	29.98
MZ191821	*V. soror*	24.36	98.57	779	30.42
NIBGE HYM-01001	*V. tropica*	34.51	71.91	658	31.00
NIBGE HYM-01572	*V. tropica*	33.91	73.39	658	31.00
BIOUG24885-F10	*V. tropica*	22.47	91.78	591	30.29
MN893829	*V. tropica*	11.07	76.86	586	30.55

**Table 2:** AIC as a criterion for model selection. GTR + Γ has the smallest AIC.

Model	lnL ^(1)^	k ^(2)^	AIC
HKY	−3092.5571	74	6333.114
HKY + Γ	−3054.0615	75	6258.123
TN93	−3083.4433	75	6316.887
TN93 + Γ	−3052.3701	76	6256.740
GTR	−3074.7981	78	6305.596
GTR + Γ	−3047.1831	79	6252.366

^(1)^ Tree log-likelihood; ^(2)^ Number of estimated parameters.

**Table 3:** Likelihood ratio tests for selecting the best substitution models. GTR + Γ is significantly better than the alternative models.

M_Special_ ^(1)^	M_General_ ^(1)^	2∆lnL ^(2)^	DF ^(3)^	*p*
HKY	TN93	18.22766	1	0.00002
HKY	GTR	35.51802	4	0.00000
TN93	GTR	17.29036	3	0.00062
HKY	HKY + G	76.99118	1	0.00000
TN93	TN93 + G	62.1463	1	0.00000
GTR	GTR + G	55.23006	1	0.00000
HKY + Γ	TN93 + G	3.38278	1	0.06588
HKY + Γ	GTR + G	13.7569	4	0.00811
TN93 + Γ	GTR + G	10.37412	3	0.01564

^(1)^ Nested special and general models (M_Special_ and M_General_); ^(2)^ Likelihood ratio chi-square statistic; ^(3)^ Degree of freedom.

## Data Availability

Data are contained within the article.
